# A Case of Vitamin D-Dependent Rickets Type 1A with a Novel Mutation in the Uzbek Population

**DOI:** 10.4274/jcrpe.3128

**Published:** 2016-12-01

**Authors:** Bahar Özcabı, Feride Tahmiscioğlu Bucak, Sevinç Jaferova, Çiğdem Oruç, Amra Adrovic, Serdar Ceylaner, Oya Ercan, Olcay Evliyaoğlu

**Affiliations:** 1 İstanbul University Cerrahpaşa Faculty of Medicine, Department of Pediatric Endocrinology, İstanbul, Turkey; 2 Intergen Genetic Center and Yüksek İhtisas University Faculty of Medicine, Department of Medical Genetics, Ankara, Turkey

**Keywords:** 25-hydroxyvitamin D 1-α hydroxylase, the CYP27B1 gene, vitamin D-dependent rickets type 1, calcitriol

## Abstract

Vitamin D-dependent rickets type 1A (VDDR-1A) (Online Mendelian Inheritance in Man #264700) is a rare, autosomal recessively inherited disorder due to inactivating mutations in CYP27B1. It is characterized by early onset of rickets with hypocalcemia. We aimed to describe the clinical and laboratory findings in a VDDR-1A case and to report a novel homozygote truncating mutation NM_000785.3 c.403C>T (p.Q135*) in CYP27B1 which to our knowledge is the first described mutation in the Uzbek population. The patient was admitted with tetany at the age of 12 months. He was a healthy Uzbek boy until 9 months of age when he had a seizure due to hypocalcemia. Vitamin D treatment was given orally in Turkmenistan (no data available for dose and duration). The patient was the product of a consanguineous marriage. His brother had died with hypocalcemia and pneumonia. At physical examination, anthropometric measurements were within normal limits; he had caput quadratum, enlarged wrists, and carpopedal spasm. Blood calcium, phosphorus, alkaline phosphatase, and parathormone (PTH) levels were 5.9 mg/dL, 3.5 mg/dL, 987 IU/L, and 182.8 pg/mL (12-72), respectively. Radiological findings included cupping and fraying of the radial and ulnar metaphyses. Renal ultrasound revealed nephrocalcinosis (grade 1). Despite high serum PTH and 25-hydroxyvitamin D3 levels, 1,25-dihydroxyvitamin D3 level was low, suggesting a diagnosis of VDDR-1A. The patient was treated with calcium carbonate and calcitriol. DNA sequencing revealed a novel homozygous mutation of NM_000785.3 c.403C>T (p.Q135*) in CYP27B1. VDDR-1A is a rare disorder which needs to be considered even in countries where nutritional vitamin D deficiency is still common.

**WHAT IS ALREADY KNOWN ON THIS TOPIC?**

Vitamin D-dependent rickets type 1A (VDDR-1A) is a rare, autosomal, recessively inherited disorder due to inactivating mutations in CYP27B1. It is characterized by early onset of rickets with hypocalcemia. In different ethnic groups, several mutations (homozygous or compound heterozygous) have been identified. Some studies reported that there is a good genotype-phenotype correlation in VDDR-1A. However, the patients carrying the mutations which can totally abolish the enzyme activity can have mild symptoms. Patients with VDDR-1A are usually treated with alfacalcidol or calcitriol.

**WHAT THIS STUDY ADDS?**

VDDR-1A is a rare disorder. We report here our clinical and treatment experience and a novel mutation in the CYP27B1 gene which as far as we know is the first described mutation in the Uzbek population.

## INTRODUCTION

Vitamin D-dependent rickets type 1A (VDDR-1A) (Online Mendelian Inheritance in Man #264700) is an inborn error of vitamin D metabolism involving defective conversion of 25-hydroxyvitamin D3 [25(OH)D3] to the active form 1,25-dihydroxyvitamin D3 [1,25(OH)_2_D_3_] by the enzyme 25(OH) D-1-hydroxylase (1). This type of rickets is characterized by hypotonia, weakness, growth failure, and hypocalcemic seizures in early infancy (1,2). The physical features, laboratory findings such as hypocalcemia with increased serum parathormone (PTH), and the radiological aspects of this condition mimic vitamin D deficiency; but typically, 25(OH)D3 levels are normal or elevated despite a low or low-normal serum 1,25(OH)2D3 level (1,2,3,4). Due to blockage of 25(OH) D-1-α-hydroxylase, treatment consists of supplementation with calcium and active forms of vitamin D (1,2).

VDDR-1A is an autosomal recessive disorder due to the mutation in the CYP27B1 gene encoding 25(OH) D-1-α-hydroxylase, which catalyzes the hormonally regulated, rate limiting step in the bioactivation of vitamin D (1,g,^3^,^4^,^5^,^6^,^7^,^8^,^9^,^10^,^11^,^12^,^13^,^14^,^15^,^16^,^17^,^18^,^19^,^20^,^21^). The CYP27B1 gene is mapped on chromosome 12q14 (4). In different ethnic groups, several mutations (homozygous or compound heterozygous) have been identified in patients with VDDR-1A (3,5,7,8,9,10,11,g,^13^,^14^,^15^,^16^,^17^,^18^,^19^,^20^,^21^). In some ethnic groups, certain mutations are more frequent (2,8,14,20). Some studies have reported that there is a good genotype-phenotype correlation in VDDR-1A (19). However, some patients carrying the mutations which can totally abolish the enzyme activity can present with mild symptoms. Additionally, partial remission during puberty may be observed more frequently in females than in males with the same mutation (8,9,19). These points lead one to speculate that there are other factors which contribute to the variations in degree of severity of the clinical and laboratory findings.

Herein, we report the clinical and laboratory findings in a case of VDDR-1A with a novel mutation NM_000785.3 c.403C>T (p.Q135*) in CYP27B1 in a boy of Uzbek origin which as far as we know is the first mutation described in the Uzbek population.

## CASE REPORT

This male patient was admitted to our hospital with the clinical symptoms of tetany at the age of 12 months. He had his first seizure in Turkmenistan when he was 9 months old; at that time, he was found to be hypocalcemic which was attributed to vitamin D deficiency as he had never received vitamin D prophylaxis. Vitamin D treatment was given orally (no data are available regarding dose and duration of treatment). His parents brought the patient to Turkey in order to get a second opinion.

The patient was the product of a consanguineous marriage and both parents were of Uzbek origin. He teethed first at the age of 10 months. He had one healthy sister and an elder brother who had died at 12 months with a history of hypocalcemia and pneumonia.

**Figure 1:**
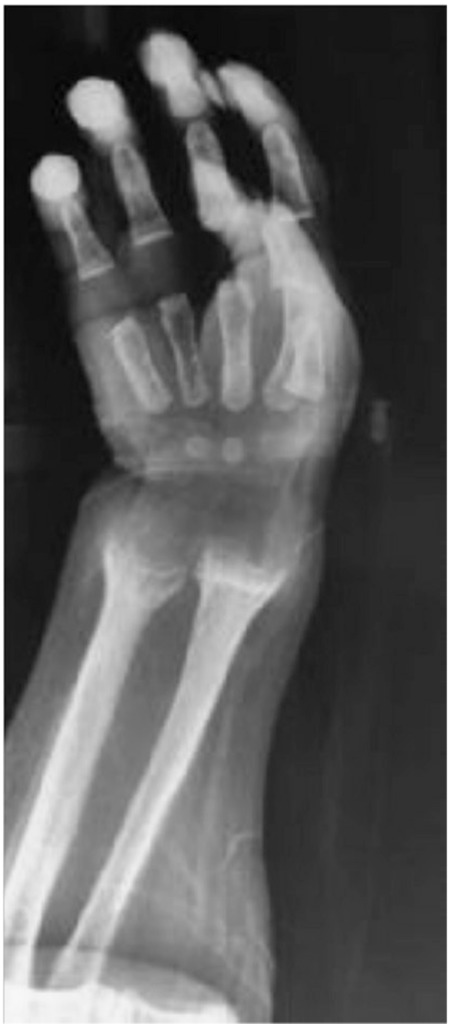
Radiological findings before treatment: cupping and fraying of the metaphyseal regions of ulna and radius (parents consented to the publication of these photos)

**Figure 2:**
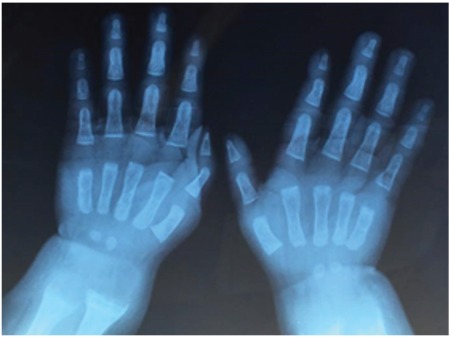
Radiological findings at the second month of the treatment: bone lesions were almost healed (parents consented to the publication of these photos)

Physical examination revealed carpopedal spasm and overactive tendon reflexes. Standard deviation score values for height, weight, and head circumference were -1.83, -1.02, and 1.64, respectively. Caput quadratum and enlargement of the wrists were distinct. He had two central incisors. Blood calcium, phosphorus, and alkaline phosphatase levels were 5.9 mg/dL (1.4 mmol/L), 3.5 mg/dL (1.15 mmol/L), and 987 IU/L, respectively. Urine calcium/creatinine ratio was 0.006 [N:<0.4 (22)] in spot sampling. Serum levels of PTH (182.8 pg/mL, N:12-72) and 25(OH)D3 levels (125 µg/L) were high and 1,25(OH)_2_D_3_ level (8.5 pg/mL, N:15-90 pg/mL) was low (Table 1). No abnormalities of acid-base metabolism and renal dysfunction were detected. Radiological findings included cupping and fraying of the metaphyseal regions of the radius and ulna. Renal ultrasonography revealed nephrocalcinosis of grade 1 (Figure 1). Clinical and laboratory findings suggested a diagnosis of VDDR-1A. Calcium carbonate (elementary calcium 75 mEq/kg/d) and calcitriol (1 µg/d) treatments were started to which the patient responded well by increasing his serum Ca level to the normal range. Laboratory findings and treatment of the patient in the follow-up are summarized in Table 1. Bone lesions were almost healed at the second month of the treatment (Figure 2).

**Figure 3:**
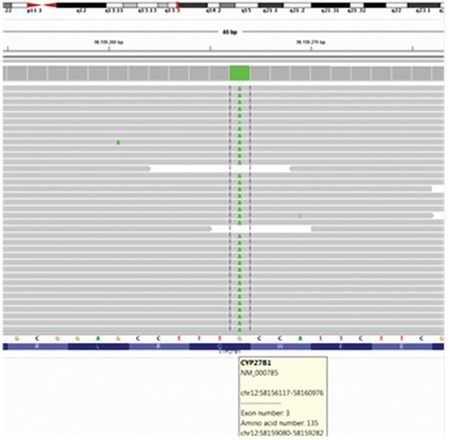
Novel mutation p.Q135* (c.403 C>T) in the CYP27B1 gene

A clinical diagnosis of VDDR-1A was made and a genetic analysis was performed to confirm the diagnosis. Sequence analysis of all coding regions and exon-intron boundaries were done by in-house designed primers using Sanger sequencing technique performed on ABI Prism 3130 GeneticAnalyser (Applied Biosystems, Inc., Foster City, CA, ABD) capillary electrophoresis system with standard protocols by using Big Dye Terminator cycle sequencing kit (Applied Biosystems, Inc., Foster City, CA, ABD) and a novel mutation NM_000785.3 (CYP27B1): c.403C>T (p.Q135*) was described (Figure 3). Mutation taster predicts this variant as a disease-causing mutation. This variant was screened in 200 healthy people and no mutation was detected in any. Other members of the patient’s family (mother, father, and elderly sister) were heterozygous carriers for this novel mutation.

## DISCUSSION

VDDR-1A is an autosomal recessive disorder due to an inactivating mutation in the CYP27B1 gene on chromosome 12q14 (1,2,3,4,5,6,7,8,9,10,11,12,13,14,15,16,17,18,19,20,21). The CYP27B1 gene encodes 25(OH) D-1-α-hydroxylase which catalyzes the hormonally regulated, rate limiting step in the bioactivation of vitamin D (1,2,3,4). Due to blockage of this enzyme activity, normal or elevated 25(OH)D3 level, despite low or low-normal serum 1,25(OH)_2_D_3_, is prominent in VDDR-1A which mimics clinically and radiologically vitamin D deficiency (1,2,3,5). There are no studies on the incidence of vitamin D deficiency in Turkmenistan where our patient lives, but there are reports from countries such as Turkey where nutritional rickets is still encountered (23). Our patient did not respond to vitamin D supplementation, but he was born to consanguineous parents and had an elder brother who was reported to have died of hypocalcemia and pneumonia. These 3 points raised suspicion and the low 1,25(OH)_2_D levels concomitant with high 25(OH)D3 levels suggested a diagnosis of VDDR-1A, which was confirmed by identification of a novel homozygous mutation of p.Q135* (c.403 C>T) in the CYP27B1.

So far, more than 50 mutations have been identified in patients with VDDR-1A from various ethnic groups (3,5,6,7,8,9,10,11,12,13,14,15,16,17,18,19,20,21). The Uzbek population is a Turkic people in Central Asia. Mutations, clinical and laboratory features of the previously reported cases with mutations in the Turkish population and the findings of our patient (presented in Table 2) indicate that age at presentation and clinical/laboratory findings show variations, even in patients with the same mutation. In our patient, the main clinical sign was hypocalcemic seizures. Also, he had hypocalcemia without hypophosphatemia. We identified a novel nonsense mutation c.403C>T (p.Q135*) in exon 3. Our patient was homozygous and other family members (mother, father, sister) were heterozygous for this novel mutation. As this mutation results in a truncating protein, it probably causes severe inactivation of the enzyme. As far as we know, this is the first mutation reported in the Uzbek population.

Patients with VDDR-1A are usually treated with alfacalcidol or calcitriol. Edouard et al (24) reported short- and long-term outcomes of calcitriol treatment in their patients. Calcitriol was started at a dose of 1.0 µg/d, given in two doses of 0.5 µg. Subsequently, the calcitriol dose was modified according to the results of biochemical analyses. Normal calcium and PTH levels without hypercalciuria were tried to be achieved. The median daily calcitriol dose was reduced to 0.50 µg/d (range 0.2-1.0 µg) after 3 months, to 0.25 µg/d (range 0.1-1.0 µg) after 1 year, and to 0.25 µg/d (range 0.1-0.5 µg) after two years of the treatment. Our patient was also treated with calcium supplementation and calcitriol of 1.0 µg/d, given in two doses. At the second month of the treatment, skeletal deformities were almost healed (Figure 2) and renal ultrasound was normal without nephrocalcinosis; but at the third month of treatment, serum phosphorus level increased to the upper normal limit and hypercalciuria was detected. The calcitriol dose was decreased to 0.75 µg/d given in three doses. At the ninth month of the follow-up, as calcitriol treatment was used in a dose higher than that prescribed (3x0.5 µg/dose instead of 3x0.25 µg/dose), serum PTH level decreased below normal ranges and urinary analysis revealed hypercalciuria. Over the next 13 months, the calcitriol dose was decreased to 0.5 µg/d, while the calcium supplementation was withdrawn at the third month of this tapering process. At the third year of follow-up, our patient was receiving 0.25 µg/d of calcitriol. His growth was normal, normocalcemia without hyperphosphatemia or nephrocalcinosis had been achieved (Table 1).

Although a rare disorder, VDDR-1A must be considered even in countries where vitamin D deficiency is still common. Genetic analyses are beneficial for early diagnosis of probable familial cases. The novel mutation NM_000785.3 c.403C>T (p.Q135*) causes a truncating protein probably associated with severe inactivity. We believe that this patient is the first case with this mutation reported in the Uzbek population.

## Ethics

Informed Consent: It was taken.

Peer-review: Externally peer-reviewed.

## Figures and Tables

**Table 1:**
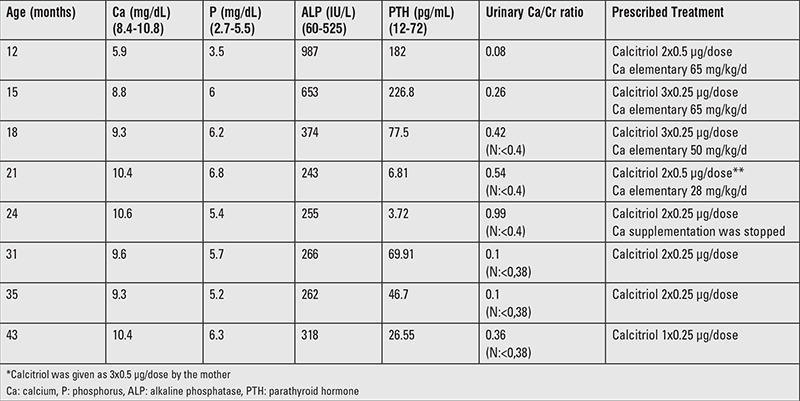
Treatment and follow-up findings of the patient

**Table 2:**
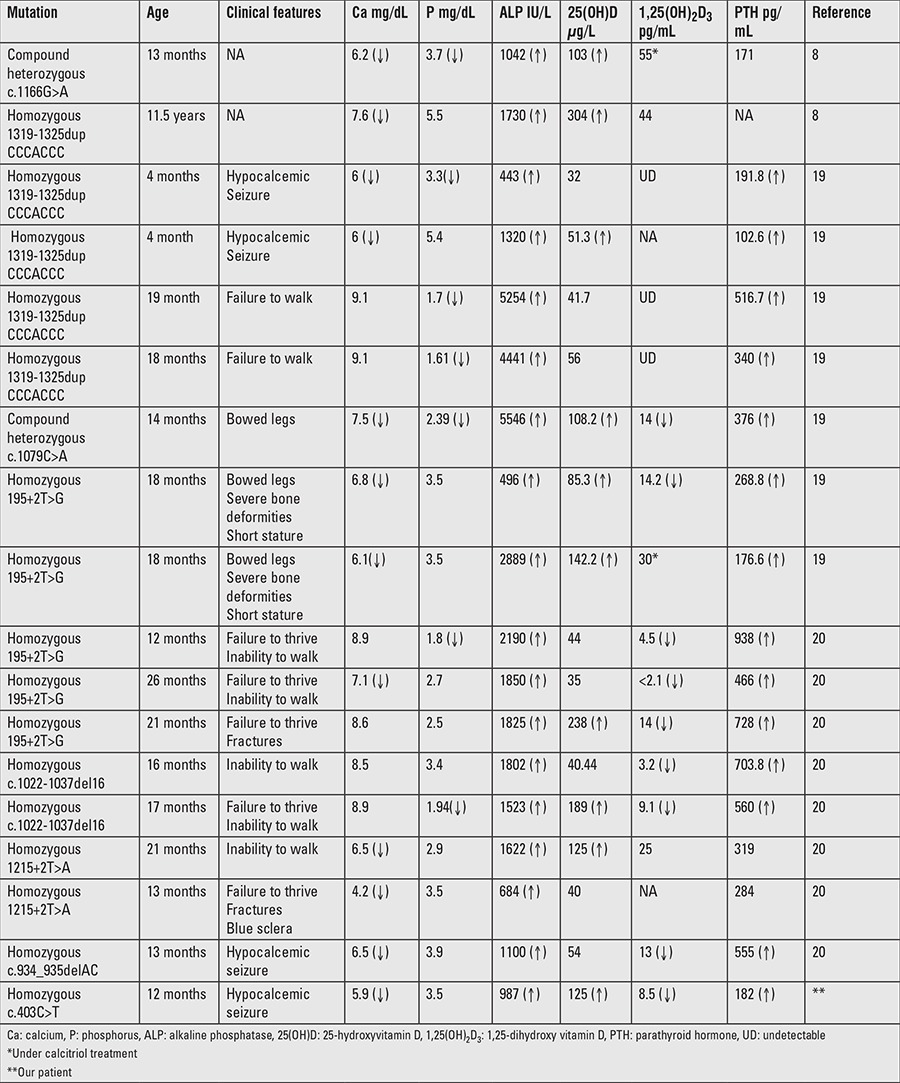
Clinical, laboratory, and genetic findings of our patient and of previously reported patients with CYP27B1 mutations in Turkish population
